# CAR T cells targeting the ganglioside NGcGM3 control ovarian tumors in the absence of toxicity against healthy tissues

**DOI:** 10.3389/fimmu.2022.951143

**Published:** 2022-08-05

**Authors:** Elisabetta Cribioli, Greta Maria Paola Giordano Attianese, George Coukos, Melita Irving

**Affiliations:** Ludwig Institute for Cancer Research, Department of Oncology, University of Lausanne and University Hospital of Lausanne Centre Hospitalier Universitaire Vaudois, Lausanne, Switzerland

**Keywords:** T cells, immunotherapy, tumors, chimeric antigen receptor (CAR), ganglioside

## Abstract

Chimeric antigen receptor (CAR) T cells have emerged as a powerful immunotherapeutic tool against certain hematological malignancies but a significant proportion of patients either do not respond or they relapse, sometimes as a result of target antigen loss. Moreover, limited clinical benefit has been reported for CAR therapy against epithelial derived solid tumors. A major reason for this is the paucity of solid tumor antigens identified to date that are broadly, homogeneously and stably expressed but not found on healthy tissues. To address this, here we describe the development and evaluation of CAR T cells directed against N-glycoslylated ganglioside monosialic 3 (NGcGM3). NGcGM3 derives from the enzymatic hydroxylation of N-acetylneuraminic acid (NAc) GM3 (NAcGM3) and it is present on the surface of a range of cancers including ovarian, breast, melanoma and lymphoma. However, while NAcGM3 is found on healthy human cells, NGcGM3 is not due to the 7deletion of an exon in the gene encoding for the enzyme cytidine monophospho-N-acetylneuraminic acid hydroxylase (CMAH). Indeed, unlike for most mammals, in humans NGcGM3 is considered a neoantigen as its presence on tumors is the result of metabolic incorporation from dietary sources. Here, we have generated 3 CARs comprising different single chain variable fragments (scFvs) originating from the well-characterized monoclonal antibody (mAb) 14F7. We show reactivity of the CAR T cells against a range of patient tumor fragments and we demonstrate control of NGcGM3^+^ SKOV3 ovarian tumors in the absence of toxicity despite the expression of CMAH and presence of NGcGM3^+^ on healthy tissues in NSG mice. Taken together, our data indicate clinical potential for 14F7-based CAR T cells against a range of cancers, both in terms of efficacy and of patient safety.

## Introduction

While T cell receptors (TCRs) recognize peptide fragments displayed on antigen presenting cells in a major histocompatibility complex (MHC)-restricted manner, in principle CARs can be designed to target any cell surface expressed antigen, including a protein, carbohydrate, glycolipid or ganglioside (1). Indeed, CARs are synthetic modular receptors comprising an antigen binding ectodomain, usually a scFv, followed by a hinge, a transmembrane region, and endodomains needed for T cell activation (i.e., CD3ζ) and co-stimulation (usually derived from CD28 or/and 41BB). Modification to CAR components, such as binding affinity of the scFv, hinge length/flexibility, and choice of costimulatory endodomain(s) (2), can have a profound impact on effector function, tumor control, and risk of on-target but off-tumor toxicity of the engineered T cells. In addition, T cell state/phenotype (3) at the time of transfer, CAR density at the cell surface, and the number of CAR T cells engrafted in proportion to tumor burden and target antigen expression levels (4), can influence the risk of adverse patient reactions. In fact, most tumor antigens targeted by CARs are also found at varying levels on healthy tissues [reviewed in (5–7)].

Major continued investments in the development of CAR T cells for treating solid tumors are predicated on the unprecedented clinical success of CD19 directed CAR T cells of up to 70-90% complete, durable responses (including some that are curative) against acute and chronic leukemias (8–11). However, CD19 represents an ideal target for CAR therapy because it is largely restricted to B cells and it is typically homogeneously expressed. Moreover, the B cells themselves can provide costimulatory support (e.g. from CD80/86) to CAR T cells, and they are readily accessible (i.e., in the bloodstream and lymphatic system) rather than being sheltered within an oftentimes difficult to access and suppressive solid tumor microenvironment (TME, 5).

Important research efforts are underway to identify solid tumor antigens that are broadly, homogeneously, and stably expressed across multiple tumor types but absent from healthy tissues (i.e., a bona fide tumor antigen rather than a tumor *associated* antigen). A deletion variant of epidermal growth factor (EGFRvIII) is an example of a tumor-restricted target (it is a driver mutation in some forms of glioblastoma), but there is considerable intra- and intertumor heterogeneity (12, 13) and antigen loss has been reported in the clinic following CAR therapy (14). Recently, proof-of-principle for the development of CARs targeting the oncogenic immunopeptidome of neuroblastoma (so-called peptide-centric CARs) has been reported (15) but, although promising, clinical efficacy and safety, as well as applicability to other cancer-types, remains to be demonstrated (16).

Here, we sought to develop CARs directed against the ganglioside NGcGM3 which we propose is a promising target tumor antigen. Gangliosides have been implicated in tumor establishment and metastases as well as in immune suppression, and numerous studies indicate that NGcGM3 is a negative prognostic marker [reviewed in (17)]. Briefly, gangliosides are glycosphingolipids having at least one sialic acid linked on the sugar chain. The two major sialic acid variants in mammals are N-acetylneuraminic acid (NAc) and N-glycolyneuraminic acid (NGc), but the latter is not found in normal human tissues due to the deletion of an exon in the gene encoding for the enzyme CMAH needed for converting NAc to NGc. Although humans lack CMAH activity, NGcGM3 derived from dietary sources (e.g., meat and dairy products) has been detected in the plasma membranes of a broad range of cancer-types including ovarian, breast, lung, melanoma, prostate, neuroblastoma, sarcoma and lymphoma as a result of their higher metabolic rate (17) and upregulation of sialin, a sialic acid transporter, by hypoxia (18). In our study, we have generated CAR T cells targeting NGcGM3 with scFv derived from the well-characterised mAb 14F7 (19, 20) and achieve *in vitro* activity against SKOV3 ovarian tumor cells as well as a range of patient biopsies. In addition, we demonstrate robust control of NGcGM3^+^ SKOV3 tumors in the absence of toxicity against healthy tissues.

## Results

### 14F7-based CAR T cells demonstrate *in vitro* activity against SKOV3 tumor cells and a panel of patient derived tumor fragments

**Figure 1:**
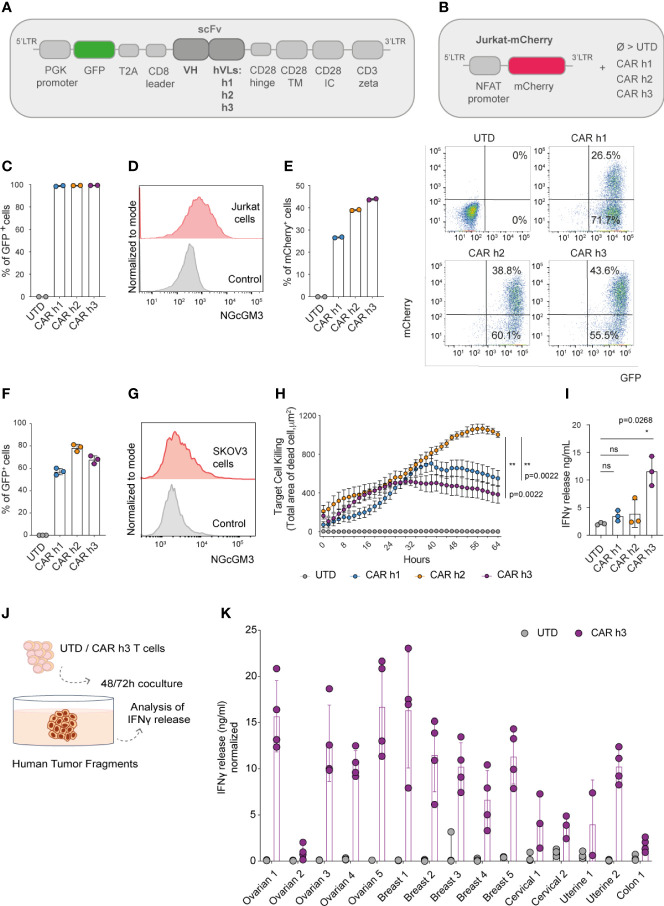
Anti-NGcGM3 CAR T cells demonstrate reactivity *in vitro* against NGcGM3^+^ SKOV3 ovarian tumor cells and a panel of patient biopsies. **(A)** Schematic of lentiviral vectors encoding GFP and the different 14F7-based anti-NGcGM3 CARs. **(B)** Schematic of the vector used to generate Jurkat-mCherry reporter cells which were then transduced with the 3 CAR variants (CAR h1, h2 and h3) or not (UTD=untransduced) **(C)** Evaluation of CAR expression by transduced Jurkat-mCherry reporter cells as assessed by GFP expression measured by flow cytometric analysis. **(D)** NGcGM3 expression on Jurkat cell surfaces as assessed by flow cytometric analysis (top, in red) and secondary antibody staining alone control (bottom, in grey). **(E)** mCherry expression levels in transduced Jurkat-mCherry reporter cells at 48h (left) and representative dot plots of reporter gene and CAR expression (evaluated by GFP expression) (right). **(F)** Percent GFP expression by lentivirally transduced primary human T cells assessed by flow cytometry. **(G)** SKOV3 cell line NGcGM3 expression (in red) assessed by flowcytometry compared to control (secondary Ab alone, in grey). **(H)** Anti-NGcGM3 CAR T cell killing of SKOV3 tumor cells (calculated as dead cell count/µm^2^) measured over days in an IncuCyte assay. **(I)** IFNγ production by anti-NGcGM3 CAR T cells upon 24h coculture with SKOV3 tumor cells. **(J)** Schematic of anti-NGcGM3 CAR untransduced (UTD) T cell coculture with patient tumor fragments. **(K)** IFNγ release in anti-NGcGM3 CAR T cell and tumor fragment coculture assays. Shown is average ± standard deviation (SD) **(F, I, K)** or standard error mean (sem) **(H)** of different cultures. Statistical analysis by unpaired, two-tailed Mann-Whitney test **(H)** and paired, two-tailed t test **(I)**. (**p< 0.01; *p < 0.05; ns, non-significant). All experiments were performed for a minimum of n=3 donors.

The IgG1 mAb 14F7 was originally generated by immunizing BALB/c mice with NGcGM3 conjugated to human very-low density lipoproteins in the presence of Freund’s adjuvant (19, 20). We began our study by generating a panel of scFv-based CARs comprising the original murine variable heavy (V_H_) domain of 14F7 and 3 previously described human variants of the variable light (V_L_) domain (herein named ‘human’ (h) h1, h2 and h3) (21) in a pRRL based lentiviral vector. Briefly, the bicistronic lentiviral transfer vectors encode the human phosphoglycerate kinase (PGK) promoter, green fluorescent protein (GFP), a T2A sequence, and the human CD8 leader sequence followed by each of the CARs [scFv, hinge, transmembrane (TM) and intracellular (IC) domains derived from CD28 and CD3ζ, **Figure 1A**].

We efficiently transduced a Jurkat-NFAT-mCherry reporter cell line (**Figure 1B**) that we previously generated (22) to express each of the 3 CARs (**Figure 1C**). Because Jurkat cells are NGcGM3^+^ *in vitro* [by NGc uptake from fetal bovine serum (FBS) in the culture media, **Figure 1D**], they quickly became activated (as evaluated by mCherry expression) following transduction to express the CARs (**Figure 1E**). As a control, the transduced reporter cells were activated for 48h with Phorbol 12-Myristate 13-Acetate/Ionomycin (PMA/Iono) (**Supplementary Figure 1A**).

Having demonstrated the ability of the 3 different CARs to trigger mCherry expression in our reporter cell line, we subsequently efficiently transduced primary human T cells derived from the peripheral blood of healthy donors (**Figure 1F**). Coculture of the human CAR T cells with SKOV3 ovarian tumor cells which are NGcGM3^+^ *in vitro* (**Figure 1G**) revealed highest target cell killing by CAR h2 engineered T cells as evaluated in an IncuCyte assay (**Figure 1H**), but highest IFNγ production by CAR h3 T cells (**Figure 1I**).

Finally, we sought to test the reactivity of anti-NGcGM3 CAR T cell against patient biopsies. We obtained a panel of ovarian, breast, cervical, uterine and colon tumor fragments, cancer-types previously shown to present NGcGM3 at their surface (17), and upon 48 or 72h coculture with CAR h3 T cells versus untransduced (UTD) T cells, we evaluated IFNγ production (**Figure 1J**). We observed varying levels of IFNγ release by the CAR h3 T cells in response to each of the tumor-types, but none by the UTD T cells (**Figure 1K**).

In summary, we built 3 different anti-NGcGM3 CARs and demonstrated *in vitro* reactivity of engineered T cells against the tumor cell line SKOV3 as well as a panel of patient biopsies. CAR h2 conferred the highest level of target cell killing and CAR h3 the highest level of IFNγ production by engineered human T cells *in vitro*.

### 14F7-based CAR T cells efficiently control the growth of NGcGM3^+^ SKOV3 ovarian tumors upon adoptive cell transfer

**Figure 2:**
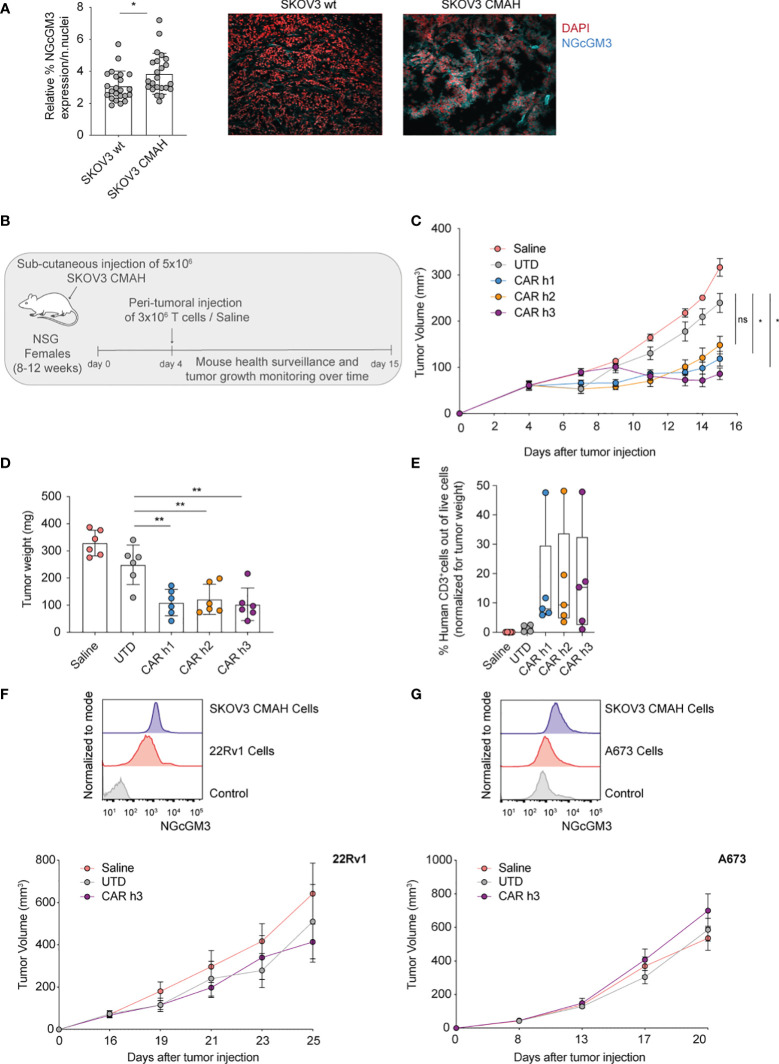
Anti-NGcGM3 CARs T cells efficiently control the *in vivo* tumor growth of an ovarian human tumor cell line expressing the target antigen. **(A)** Relative expression of NGcGM3 on SKOV3 wt versus SKOV3 CMAH subcutaneous tumor fragments (left). Representative immunofluorescence images (right); DAPI staining of nuclei in red, 14F7 mAb plus Alexafluor 647 labeled secondary Ab in blue. **(B)** Schematic of *in vivo* ACT study. **(C)** Tumor growth (SKOV CMAH) curves over days following subcutaneous injection. **(D)** Tumor weights (SKOV3 CMAH) at the end of the study (day 15). **(E)** Percentage of human CD3^+^ T cells infiltrating tumors at the end of the study (normalized for tumor volume). **(F, G)** Relative expression levels of NGcGM3 on SKOV3 CMAH, 22Rv1 and A673 tumor cells assessed by flow cytometry (control = secondary Ab alone) (top). Tumor growth curves for 22Rv1 and A673 over days (in this ACT study the mice received 2x10^6^ CAR T or UTD cells by peritumoral injection at days 16 and 8, respectively, post tumor injection) (bottom). Shown is average ± SD **(A, D)** or ± sem **(C)** and (**F**, **G**, bottom panels) or box and whiskers (min to max) **(E)**. Statistical analysis by unpaired, two-tailed Mann-Whitney test **(A)** and paired, two-tailed t test **(D)**; two-way analysis of variance (ANOVA) with correction for multiple comparisons by *post hoc* Tukey’s test **(C)**; unpaired, two-tailed t test **(D)**.(**p< 0.01; *p < 0.05; ns, non-significant).

The human ovarian SKOV3 cell line has been previously gene-modified to express CMAH (named SKOV3 CMAH) needed for the enzymatic hydroxylation of NAcGM3 to NGcGM3, and it has been shown that intraperitioneal administration of humanized 14F7 mAb efficiently controls SKOV3 CMAH growth *in vivo* (23). With the aim of evaluating our anti-NGcGM3 CAR T cells *in vivo*, we began by analysing NGcGM3 expression by wild type (wt) SKOV3 versus SKOV3 CMAH subcutaneous tumors *ex vivo* and confirmed elevated expression levels by the latter (**Figure 2A**).

For *in vivo* testing of the anti-NGcGM3 CAR T cells we subcutaneously engrafted NSG mice with SKOV3 CMAH cells which are able to convert NAcGM3 to NGcGM3 (schematic shown in **Figure 2B**). Because CMAH is expressed in murine cells we transferred the T cells by peritumoral injection to avoid systemic on-target but off-tumor toxicity, or/and sequestration of the CAR T cells in healthy tissues. We measured significant tumor control by CAR h1 and h3 T cells as compared to treatment with UTD T cells and saline alone (**Figure 2C**). Evaluation of tumors at the end of the study revealed a similar and significant reduction in weight (**Figure 2D**) and comparable T cell infiltration levels upon treatment with the 3 different CARs as compared to the controls (**Figure 2E**).

In two additional independent *in vivo* experiments with lower doses of CAR h3 T cells we demonstrated significant tumor control, a significant reduction in tumor weight at the end of the study, and confirmed significant CAR h3 T cell infiltration, as compared to UTD T cells (**Supplementary Figures 1B–** **G**). We further evaluated CAR h3 T cells *in vivo* against prostate (22Rv1) and Ewing's sarcoma (A673). While both of these cell lines uptake and present NGcGM3 *in vitro* (from FBS in the culture medium) (**Figures 2F**, **G**, top) we did not observe any tumor control *in vivo*. This is not unexpected because these human tumor cell lines do not express CMAH and the mice receive a vegetarian diet (i.e., there is not the possibility of NGcGM3 uptake by the tumors *in vivo*).

In summary, we demonstrated significant control of SKOV3 CMAH ovarian tumors upon adoptive transfer of 14F7-based anti-NGcGM3 CAR T cells, but not of 22Rv1 prostate nor A673 sarcoma tumors.

### Anti-NGcGM3 CAR T cells do not cause toxicity against healthy tissues in NSG mice

NSG mice express the enzyme CMAH thus healthy murine cells can present enzymatically generated NGcGM3 in their outer membranes. However, while humans do not express CMAH, one cannot exclude the possibility of dietary uptake of NGcGM3 by normal tissues. Hence, we sought to evaluate the potential for anti-NGcGM3 CAR T cell toxicity against healthy tissues in NSG mice as a surrogate for potential toxicity against human tissues arising from dietary uptake of NGcGM3 (24).

**Figure 3:**
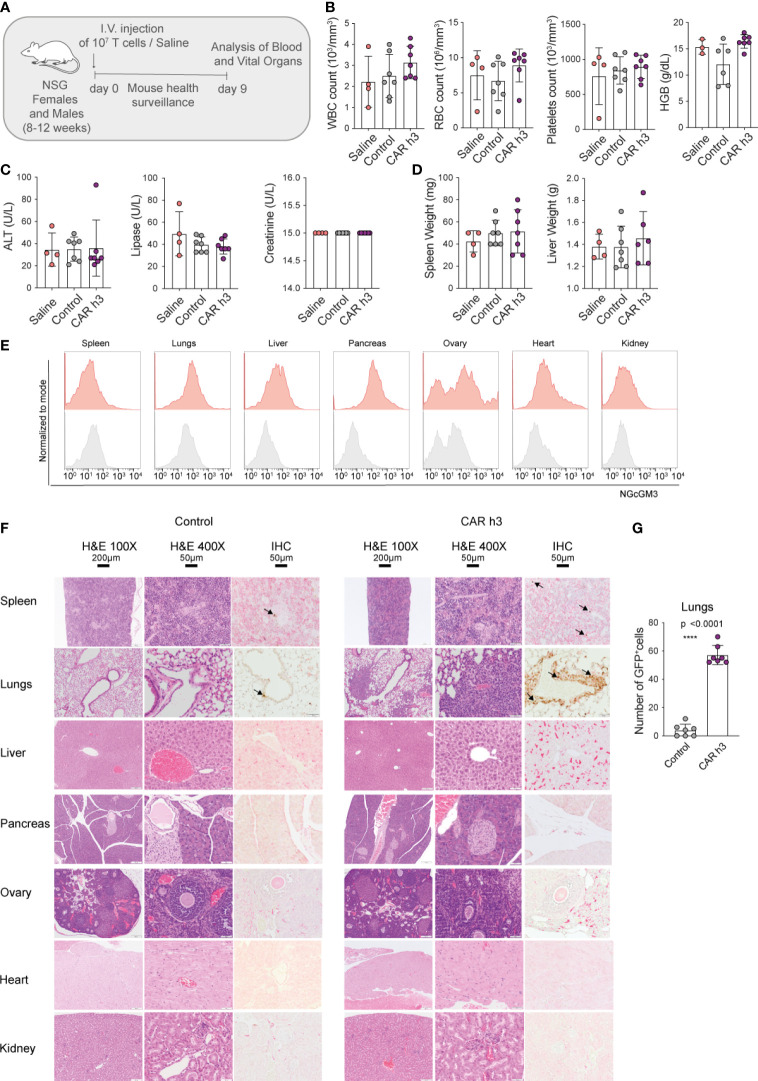
Anti-NGcGM3 CAR T cells do not cause toxicity in NSG mice. **(A)** Schematic of *in vivo* toxicity study. **(B)** Blood analysis to assess white blood cells (WBC), red blood cells (RBC), platelets counts and hemoglobin (HGB). **(C)** Measurement of alanine aminotransferase (ALT), lipase and creatinine in serum. **(D)** Spleen and liver weight upon necropsy. **(E)** NGcGM3 expression in different organs (spleen, lungs, liver, pancreas, ovary, heart, kidney). **(F)** Histopathology of organs from control (GFP transduced T cells) and anti-NGcGM3 CAR h3 T cell treated mice on day 9 post intravenous injection of transduced 10^7^ T cells. H&E = hematoxylin and eosin staining at 100X and 400X magnification; IHC = immunohistochemistry to detect GFP^+^ T cells (brown, indicated with arrow). **(G)** Quantification of GFP^+^ T cells in the lungs. Shown is average ± SD **(B, C, D, G)**. Statistical analysis by unpaired, two-tailed t test **(G)**. (****p< 0.0001).

We intravenously injected both female and male NSG mice with a high dose of anti-NGcGM3 CAR h3 T cells (10^7^), as well as control GFP^+^ T cells and saline, and carefully monitored them for 9 days (schematic in **Figure 3A**). We observed no weight loss of the mice (**Supplementary Figure 2A**) nor signs of distress. In addition, hematocrit analysis of the blood 8 days post-adoptive cell transfer (ACT) revealed no difference in the levels of white blood cells (WBC), red blood cells (RBC), platelets, or systemic hemoglobin (HGB) levels amongst the CAR T cell treated versus control mice (**Figure 3B**, left to right). Similarly, analysis of the sera indicated no signs of liver, pancreatic or kidney toxicity as there were no differences in alanine aminotransferase (ALT), lipase, and creatinine levels respectively, amongst the groups of mice (**Figure 3C**, left to right). At autopsy we observed no difference in liver or spleen weights for the CAR T cell treated versus control mice (**Figure 3D**, left and right). Flow cytometric analysis of 14F7 mAb stained single cell suspensions of organs revealed varying levels of NGcGM3 expression for the spleen, lungs (even though lower in comparison to SKOV3 CMAH tumor cells; **Supplemental Figure 2B**), liver, pancreas, ovary, heart and kidney (**Figure 3E**, left to right), and substantially lower NGcGM3 levels on the brain and prostate (**Supplementary Figure 2C**, left and right).

Finally, by histopathology (blinded study by a trained pathologist) we observed no signs of toxicity by the anti-NGcGM3 CAR T cell treatment to the spleen, lungs, liver, pancreas, ovary, heart and kidney (**Figure 3F**, top to bottom), or to the brain and prostate (**Supplementary Figure 2D**). Constitutive GFP expression by both the control and CAR h3 T cells allowed for semi-quantitative analysis of the transferred T cells. We observed higher levels of CAR h3 T cells versus control T cells in the lungs (in the absence of any aberration of the vessels or alveolar walls; **Figure 3F**, **G**), in line with previous reports of activated T cell retention in the lungs upon intravenous transfer (25). Notably, anti-F4/80 staining of the different organ cross-sections revealed no differences in macrophage infiltration into organs (except for the lungs) for the CAR T cell versus control mice T cells treated (**Supplementary Figure 2E**), further indicative that no toxicity was caused by the treatment. In an independent ACT study, we confirmed the daily well-being of the mice upon high doses of anti-NGcGM3 CAR T cell transfer, and once again showed higher retention of both CD8^+^ and CD4^+^ CAR T cells in the lungs of mice (**Supplementary Figure 2F**).

In summary, despite the expression of CMAH in NSG mice, and the presence of NGcGM3 on most organs, transfer of high doses of CAR h3 T cells did not cause adverse reactions against healthy tissues.

## Discussion

The unprecedented clinical success of CAR T cells against some advanced hematological malignancies has driven tremendous efforts to develop effective CAR therapies for treating epithelial derived solid tumors which represent the majority of cancers. Obstacles to solid tumor control by CAR T cells can be divided into 3 main categories: (i) insufficient CAR T cell homing and infiltration, (ii) barriers in the TME that are limiting to CAR T cell persistence and effector function, and, (iii) the paucity of target tumor antigens that are broadly, homogeneously and stably expressed but not found on healthy tissues. Here, we sought to address the identification of suitable solid tumor antigen targets for CAR therapy.

In our study, we explored NGcGM3 as a CAR target. NGcGM3 is a ganglioside that is not endogenously produced in humans due to the deletion of an exon in the gene encoding for the enzyme CMAH required for the conversion of NAc to NGc. However, as a result of dietary uptake by highly metabolic tumor cells, NGcGM3 has been identified as present on a range of human tumors, both epithelial cell derived and of hematological origin. NGcGM3 levels can range from moderate to intense depending on tumor-type and the patient [reviewed in (17)]. To build our CAR panel, we took advantage of the previously described murine mAb 14F7 which can exquisitely distinguish NGcGM3 from NAcGM3 *via* a subtle chemical modification of a CH_2_OH group instead of CH_3_ in the context of a trisaccharide. Notably, the anti-NGcGM3 mAb 14F7 labeled with (99m)Tc has been used to demonstrate clinical evidence of NGcGM3 expression in human breast cancer (26) and the GlycoVaxGM3 vaccine, a nanoparticulated product obtained through the insertion of NGcGM3 into the outer membrane protein complex of *N. meningitides*, has been tested in the clinic (27–29).

We successfully built second generation CARs comprising 3 different scFvs targeting NGcGM3. The scFvs that we employed comprise the original murine V_H_ of 14F7 linked to 3 different human V_L_ fragments previously identified by phage display light chain shuffling (21). All 3 of our CARs conferred *in vitro* reactivity of engineered human T cells against SKOV3 tumor cells. Moreover the best CAR candidate showed functional activity against a panel of human tumor fragments. In addition, we demonstrated *in vivo* tumor control of NGcGM3^+^ SKOV3 ovarian tumors in the absence of any toxicity against healthy tissues in NSG mice, despite the observed presence of NGcGM3 across many organs. Notably, ovarian cancer is the 8^th^ most commonly diagnosed cancer in women globally, and it is the 4^th^ most common cause of cancer-related death in women in the developed world. Indeed, due to the lack of specific symptoms, nearly 75% of ovarian cancer patients are diagnosed at a late stage with widespread intra-abdominal disease (30) and an effective CAR therapy would thus represent an important medical breakthrough (31).

The mAb 14F7 has been extensively characterized with respect to its specific reactivity against NGcGM3 (17) but concerns have been raised about potential reactivity against healthy tissues in which NAcGM3 may be naturally present at high levels, as well as NGcGM3 from dietary sources. In our study, we detected NGcGM3 on human Jurkat (T cell leukemia), SKOV3 (ovarian), 22Rv1 (prostate) and A673 (Ewing’s sarcoma) tumor cell lines *in vitro*, most probably acquired from the FBS [an abundant source of NGcGM3 (18, 32)] in the culture medium. Similarly, others have reported NGcGM3 on retinoblastoma (33) and epidermoid carcinoma (34) cell lines *in vitro*. However, *in vivo* we achieved tumor control of SKOV3 CMAH tumors (i.e., overexpressing the enzyme needed to generate NGc from NAc) but not of 22Rv1 or of A673 tumors. Indeed, because the NSG mice receive vegetarian nourishment there is no dietary source of NGcGM3 for the tumors to acquire. Notably, in our ACT study in which NSG mice received 10^7^ anti-NGcGM3 CAR T cells there were no signs of toxicity identifiable in the blood or to any of the organs despite that they express NGcGM3. Taken together, these observations indicate that there is a minimum threshold of NGcGM3 that must be present for 14F7-based CAR T cell reactivity. If change to diet (i.e., high consumption of meat or dairy products) can increase anti-NGcGM3 CAR T cell responses against tumors and/or lead to toxicity against healthy tissues has not been explored in this study but is relevant to their clinical translation (6).

As described above, we employed 14F7 derived scFvs comprising a murine V_H_ region and human V_L_ regions. Such murine/human scFv could potentially be immunogenic in humans resulting in unwanted depletion of the CAR T cells (35). The mAb 14F7 has been humanized (14F7hT) to reduce its immunogenicity (36), and the testing of fully humanized scFv variants for CAR therapy is warranted. Of course, any new scFv should be carefully evaluated for retained target specificity, as well as for propensity to aggregate at the T cell surface which can result in tonic signaling and T cell exhaustion (37, 38). Notably, 14F7hT has been demonstrated by others to exhibit significant antitumor effects in preclinical hematological tumor models [reviewed in (17)]. Indeed, anti-NGcGM3 CAR T cells offer the possibility of treating a range of solid and liquid tumors alike.

Another approach to improve the efficacy of anti-NGcGM3 CAR T cells is to co-engineer them with gene-cargo that can either directly support the fitness/function of the CAR T cells themselves or/and reprogram the TME to harness endogenous immunity (39). We have comprehensively demonstrated, for example, the numerous benefits of IL-15 coengineering of murine CAR T cells in a syngeneic melanoma tumor model (40). However, the impact of additional gene-cargo on target tumor antigen must be carefully evaluated. For example, although transgenic expression of IL-15 was shown to improve the antiglioma activity of IL-13alpha2 CAR T cells, antigen loss was reported (41).

Because of the broad expression of NGcGM3 by both solid and liquid tumors *via* dietary uptake in humans, there is also the potential for coadministration of anti-NGcGM3 CAR T cells with CAR T cells targeting a second antigen as a means of mitigating escape (42). Or, one could develop anti-NGcGM3 costimulatory CARs to enhance T cell receptor (TCR) based immunotherapy (43), or in the context of a parallel (p)CAR design (44). Finally, in recent years, several remote-control designs including ON-CARs (45), STOP-CARs (22), and OFF-CARs (46, 47) have been developed that could provide the means to more safely explore the translation of NGcGM3 redirected T cells to the clinic. Taken together, we conclude from our study and recent literature the strong clinical potential of NGcGM3 redirected CAR T cells for immunotherapy against a broad range of cancers.

## Materials and methods

### 14F7-based CAR construction

Second generation self-inactivating (SIN) lentiviral expression vector pRRL containing single chain fragment variable specific for PSMA (22) was used as a starting construct for building the second generation antiNGcGM3 CARs. Three human variants of the V_L_ (7Ah, 8Bh and 7Bh, which we named CAR h1, CAR h2 and CAR h3), previously developed (21), were ordered as genestrings (GeneArt, Invitrogen) and cloned in the lentiviral vector using SpeI and SalI restriction site digestion in frame with CD28 derived hinge, TM, IC domains and a CD3ζ signaling endodomain, under the control of a human PGK promoter, in a bicistronic construct together with the gene reporter GFP. The two proteins are separated by T2A self cleaving peptide. For toxicity *in vivo* experiments, control T cells were transduced with a pRRL vector carrying GFP only to allow the *ex vivo* tracing of transferred cells.

### Recombinant lentivirus production

All plasmids were purified using the HiPure Plasmid Filter Maxiprep Kit (Invitrogen, Thermo Fisher Scientific). High-titer replication-defective lentivirus were produced and concentrated for primary T cell transduction. Briefly, 24h before transfection, 293T human embryonic kidney (HEK) cells were seeded at 10^7^ in T-150 tissue culture flasks. HEK cells were transfected with pVSV-G (VSV glycoprotein expression plasmid), R874 (Rev and Gag/Pol expression plasmid), and pRRL transgene plasmid using a mix of Turbofect (Thermo Fisher Scientific AG) and Optimem media (Invitrogen, Lifetechnologies). The viral supernatant was harvested at 48h post-transfection. Viral particles were concentrated for 2h at 24,000g at 4°C with a Beckman JS-24 rotor (Beckman Coulter) and resuspended in fresh culture media followed by immediate snap freezing in dry ice.

### Human T cell transduction and expansion

Primary human T cells were isolated from the peripheral blood mononuclear cells (PBMCs) of healthy donors (HDs) prepared as buffy coats. All blood samples were collected with informed consent of the donors. Total PBMCs were obtained *via* Lymphoprep (Axonlab) separation solution using a standard protocol of centrifugation. CD4^+^ and CD8^+^ T cells were isolated using a negative selection kit coupled with magnetic beads separation (easySEP, Stemcell Technology). T cells were then cultured in complete media [RPMI 1640 with Glutamax, supplemented with 10% heat-inactivated FBS (Gibco), 100 µg/ml penicillin, 100 U/ml streptomycin sulfate (Invitrogen, Lifetechnologies)], and stimulated with anti-CD3 and anti-CD28 mAb coated beads (Lifetechnologies) in a ratio of 1:2, T cells: beads. Twelve to 24h after activation, T cells were transduced with lentivirus particles titrated by serial dilution in Jurkat cells. CD4^+^ and CD8^+^ T cells used for *in vitro* experiments were mixed at a 1:1 ratio, activated, and transduced. For *in vivo* studies and *in vitro* coculture with tumor fragments, CD4^+^ and CD8^+^ T cells were activated, transduced separately and then mixed prior to the experiments at a 20%: 80%, CD4^+^: CD8^+^ ratio. Human recombinant IL-2 (h-IL-2; Peprotech) was added every other day to obtain a 50 IU/ml final concentration until 5 days post stimulation (day +5). At day +5, magnetic beads were removed and h-IL-2 was switched to h-IL-15 and h-IL-7, both at 10 ng/mL (Miltenyi Biotec GmbH). A cell density of 0.5-1 × 10^6^ cells/ml was maintained for expansion. Rested engineered T cells were adjusted for identical transgene expression before all functional assays.

### Cell lines

293T HEK, Jurkat, 22Rv1 and A673 cells were purchased from the ATCC. SKOV3 wt and SKOV3 CMAH were kindly provided by Dr. Kalet Leon (CIM, Cuba). The Jurkat-mCherry cell line generated in the lab was engineered to express a 6x NFAT-mCherry -reporter system such that upon activation the cells turn red. 293T HEK, 22Rv1, A673 and Jurkat cells were cultured in complete media. SKOV3 wt and SKOV3 CMAH were cultured in DMEM supplemented with 10% heat-inactivated FBS, 2 mmol/l L-glutamine, and 100 µg/ml penicillin, 100 U/ml streptomycin. To select SKOV3 CMAH^+^ cells, geneticin (Invitrogen G418, 1-2 mg/mL) was added to the culture medium.

### Cytokine release assays

Cytokine release assays were performed by co-culture of 5x10^4^ T cells with 5x10^4^ target cells per well, in duplicate, in 96-well round bottom plates in a final volume of 200µl complete media. After 24h, co-culture supernatants were harvested and tested for presence of human IFN-γ using an ELISA Kit, according to the manufacturer’s protocol (Biolegend). The reported values represent the mean of engineered T cells derived from three HDs. Patients derived tumor fragments were sectioned in 2-3 mm cubes and cocultured with T cells in a 96 well round bottom plate for 48-72h prior to supernatant collection and IFN-γ release analysis with ELISA (Biolegend).

### Cytotoxicity assays

Cytotoxicity assays were performed using the Incucyte System (Essen Bioscience). Briefly, 1.25×10^4^ target cells were seeded 18h before the co-culture set up in flat bottom 96 well plates (Costar, Vitaris). The following day, rested T cells (no cytokine addition for 48h) were counted and seeded at 2.5x10^4^/well, at a ratio 1:2, target:T cells in complete media. No exogenous cytokine was added in the assay medium during the co-culture period. Cytotox Red reagent (Essen Bioscience) was added at a final concentration of 125nM in a total volume of 200µl. Images of total number of red cells/μm^2^ were collected every 2h of the co-culture for a total of 3 days and were analyzed using the software provided by the Incucyte manufacturer. Data are expressed as mean of 3 different HDs +/- standard deviation.

### Flow cytometric analysis

InfraRed live/dead was used for viability staining. All mAbs were purchased from BD Biosciences. Tumor cell surface expression of NGcGM3 was achieved by primary staining with 14F7 mAb (kindly provided by Dr. Kalet Leon, Cuba) and then secondary staining with Alexafluor 647 anti-mouse Fc mAb. (115-605-071, Jackson Immune research Laboratory) Acquisition and analysis were performed using a BD FACS LRSII with FACS DIVA software (BD Biosciences).

### Immunohistochemistry

Wild type SKOV3 and SKOV3 CMAH subcutaneous tumors were cryopreserved in OCT compound prior to sectioning (Mouse Pathology Facility, University of Lausanne) and staining. The tumors sections were fixed with a solution of 10% NBF (Formalin solution neutral buffered, HT501128, Sigma), permeabilized with a solution of PBS 0.5% Triton (X100, Sigma), and the aspecific binding sites were blocked with a solution of PBS, 2% heat-inactivated FBS and 1% BSA. The samples were then incubated overnight with 10μg/ml 14F7 mAb. Upon extensive washes with PBS the tumor sections were incubated with secondary Ab anti- antigen binding fragment (Fab) labeled with Alexafluor 647 (115-606-072, Jackson Immune research Laboratory) for 1h at RT. The sections were further stained with DAPI (D9545, Sigma Aldrich) and the slides then analyzed with an Epifluorescence microscope.

### Jurkat-NFAT-mCherry cell line transduction and reporter assays

Jurkat-NFAT-mCherry reporter cells previously developed in the lab (22) were transduced with lentivirus encoding both GFP and each of the different anti-NGcGM3 CARs. Briefly, 1x10^6^ cell/mL cells were seeded into 48-well plates in 500 µL/well and 50 µL of virus supernatant was mixed with protamine sulfate (P4020, Sigma Aldrich) for a final concentration of 10 µg/mL. After incubation for 24h at 37°C the cell media was refreshed and the cells were incubated for an additional 72h at 37°C before use. The transduced cells were cultured with the addition or not of PMA/lono for 48h and analyzed by flow cytometry for mCherry expression (FL-2 channel) and GFP (FL-1 channel).

### Mice and *in vivo* experiments

NOD SCID gamma knock-out (NSG) mice were bred and housed in a specific and opportunistic pathogen-free (SPF) animal facility at the Epalinges campus of the University of Lausanne. All experiments were conducted according to the Swiss Federal Veterinary Office guidelines and were approved by the Cantonal Veterinary Office. All cages housed 5 animals in an enriched environment providing free access to food and water. During experimentation, all animals were monitored at least every other day. Mice were euthanized upon meeting distress criteria and at end-point by carbon dioxide overdose. A total of 5x10^6^ SKOV3 wt or 5x10^6^ SKOV3 CMAH tumor cells were subcutaneously injected in flanks of mice (6-10 mice per group). Tumor volume was monitored by caliper measurements every other day starting from day 4 post injection. For ACT experiments, 2-10 x10^6^ CAR^+^ T cells (CD8^+:^ CD4^+^ = 80%: 20%) were peritumorally or intravenously injected and tumor volume was monitored over time as indicated. Tumor volume was determined with the calculation: volume in mm^3^ = (length x width^2^)/2, where length is the greatest longitudinal measurement and width is the greatest transverse measurement.

### *In vivo* toxicity study

To evaluate *in vivo* toxicity of anti-NGcGM3 CAR T cells, NSG mice received an intravenous injection of 10^7^ transduced T cells (control GFP, CAR). The mice were monitored daily for 9 days at which time point they were sacrificed and the organs collect (blood was sampled on day 8).

The blood was analyzed in a blinded manner for white blood cell (WBC) count, red blood cell (RBC) count, platelets and hemoglobin (HGB) concentration with the mythic18 Vet instrument according to the manufacturer’s suggestions. The serum was analyzed for units per liter (U/L) of alanine aminotransferase (ALT), lipase, and creatinine at the Clinical Chemistry Laboratory at the Lausanne University Hospital (CHUV).

For Haematoxylin-Eosine (H&E) staining, 4µm paraffin sections were stained using a standard histology procedure to assess general morphology. For the double IHC F4/80 and GFP staining, the double chromogenic IHC assay was performed using the Ventana Discovery ULTRA automate (Roche Diagnostics, Rotkreuz, Switzerland). All steps were performed automatically with Ventana solutions. Primary mAbs were applied sequentially. First, dewaxed and rehydrated paraffin sections were incubated with a rat anti-F4/80 mAb (clone Cl:A3-1, Thermo Fisher MA191124, diluted 1:50), followed by a rat Immpress AP (Vector Laboratories) and revelation with the Discovery red chromogen. Next, a heat pretreatment was applied using the CC1 solution for 40 min at 95°C. Sections were subsequently incubated with a goat anti-GFP mAb (Abcam ab6673, diluted 1:400), followed by a goat Immpress HRP (Vector Laboratories) and revelation using the ChromoMap DAB chromogen. Sections were counterstained with Mayer hematoxyline (J.T. Baker) and permanently mounted with Pertex (Sakura). All H&E stainings were performed in a at the Histology Core Facility at the Swiss Federal Institute of Technology in Lausanne (EPFL). Slides were analyzed in a blinded manner by a trained pathologist at the same facility.

### Statistical analysis

GraphPad Prism 9.0 (GraphPad Software, La Jolla, CA) was used for statistical calculations. P < 0.05 was considered significant. Statistical analyses used include two-way ANOVA, unpaired two-tailed Mann-Whitney, and two-tailed paired and unpaired t tests, depending on the type of experiment and as indicated in the figure legends.

## Data availability statement

The raw data supporting the conclusions of this article will be made available by the authors, without undue reservation.

## Ethics statement

The animal study was reviewed and approved by Swiss Cantonal Veterinary Office.

## Author contributions

MI directed the study and GC provided expert advice. EC, GMPGA and MI planned experiments and interpreted results. EC and GMPGA performed experiments. EC, MI and GMPGA wrote the paper. All authors contributed to the article and approved the submitted version.

## Funding

Ludwig Cancer Research, The Swiss National Science Foundation (SNSF/FNS to MI: 310030_204326), The Prostate Cancer Foundation and Cancera.

## Conflict of interest

The authors declare that the research was conducted in the absence of any commercial or financial relationships that could be construed as a potential conflict of interest.

## Publisher’s note

All claims expressed in this article are solely those of the authors and do not necessarily represent those of their affiliated organizations, or those of the publisher, the editors and the reviewers. Any product that may be evaluated in this article, or claim that may be made by its manufacturer, is not guaranteed or endorsed by the publisher.

## Supplementary material

Supplementary Figure 1
**(A)** Evaluation by flow cytometry of GFP and mCherry expression by Jurkat NFAT-mCherry reporter cells transduced or not with anti-NGcGM3 CARs and stimulated with phorbol myristate acetate and ionomycin (PMA/Iono). **(B)** SKOV3 CMAH tumor growth curves over days upon peritumoral injections of 2x10^6^ T cells (versus 3x10^6^ T cells in **Figure 2B** to test the activity and infiltration of a lower number of T cells) or saline. **(C)** Tumor weight at the end of the study. **(D)** Percentage of human CD3^+^ T cells and **(E)** CD3^+^GFP^+^ cells infiltrating tumors at the end of the study (normalized for tumor volume). **(F)** SKOV3 CMAH tumor growth curves over days upon peritumoral injections of 2x10^6^ T cells or saline (independent repetition of B). **(G)** Tumor weight at the end of the study. Shown is average ± sem **(B, F)**, ± SD **(C, G)** or box and whiskers (min to max) **(D)**. Statistical analysis by two-way ANOVA **(B, F)** and unpaired two-tailed t test **(C, D, G)**. (****p< 0.0001, **p< 0.01; *p < 0.05).

Supplementary Figure 2
**(A)** Weight of female and male mice over time for *in vivo* toxicity study (treated as per **Figure 3A**). **(B)** Flow cytometric analysis to evaluate NGcGM3 expression by 14F7 mAb staining of dissociated lungs (in purple) and SKOV3 CMAH cells (in red) compared to control (secondary Ab alone, in grey). **(C)** Flow cytometric analysis to evaluate NGcGM3 expression by 14F7 mAb staining for different dissociated organs (in red) compared to control (secondary Ab alone, in grey). **(D)** Histopathology of the organs [treated as per **Figure 3A**): H&E = hematoxylin and eosin staining at 100X and 400X magnification; IHC = immunohistochemistry to detect GFP^+^ T cells (brown, indicated with arrow)]. **(E)** Quantification of GFP^+^ and F4/80^+^ cells in different organs evaluated by IHC (treated as per **Figure 3A**). **(F)** Flowcytometric analysis of dissociated organs and peripheral blood (mice treated as per **Figure 3A**) to detect human CD8^+^ **(left)** and CD4^+^ **(right)** T cells. Shown is average ± sem **(A)**, ± SD **(E)**. Statistical analysis unpaired two-tailed t test **(E)**. (****p< 0.0001).
