# Supplementary figures and images for: CAR T cells targeting the ganglioside NGcGM3 control ovarian tumors in the absence of toxicity against healthy tissues
Source: Front Immunol. 2022 Aug 5;13:951143. doi: 10.3389/fimmu.2022.951143 (PMC9389107; doi:10.3389/fimmu.2022.951143)

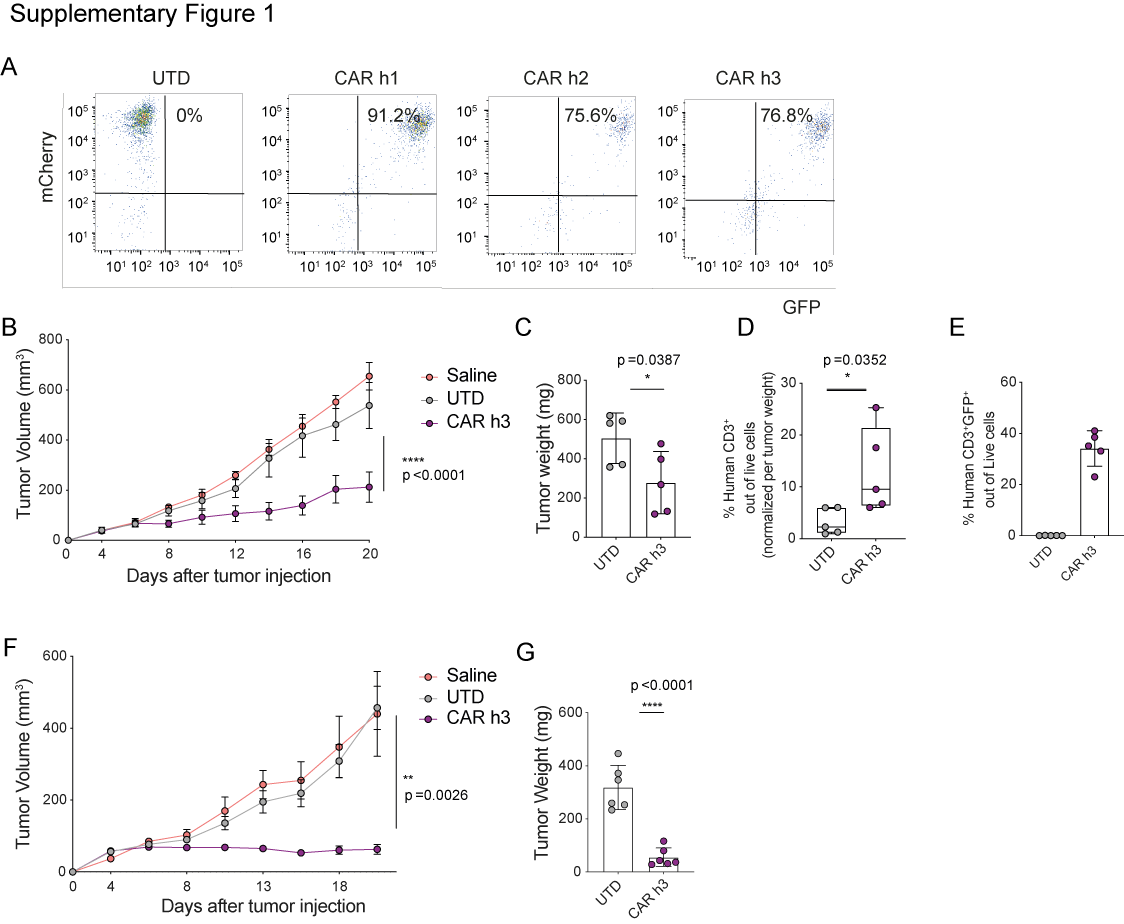

Supplement: Supplementary Figure 1 — (A) Evaluation by flow cytometry of GFP and mCherry expression by Jurkat NFAT-mCherry reporter cells transduced or not with anti-NGcGM3 CARs and stimulated with phorbol myristate acetate and ionomycin (PMA/Iono). (B) SKOV3 CMAH tumor growth curves over days upon peritumoral injections of 2x10^6^ T cells (versus 3x10^6^ T cells in Figure 2B to test the activity and infiltration of a lower number of T cells) or saline. (C) Tumor weight at the end of the study. (D) Percentage of human CD3+ T cells and (E) CD3+GFP+ cells infiltrating tumors at the end of the study (normalized for tumor volume). (F) SKOV3 CMAH tumor growth curves over days upon peritumoral injections of 2x10^6^ T cells or saline (independent repetition of B). (G) Tumor weight at the end of the study. Shown is average ± sem (B, F), ± SD (C, G) or box and whiskers (min to max) (D). Statistical analysis by two-way ANOVA (B, F) and unpaired two-tailed t test (C, D, G). (****p< 0.0001, **p< 0.01; *p < 0.05). [file Image_1.tif]

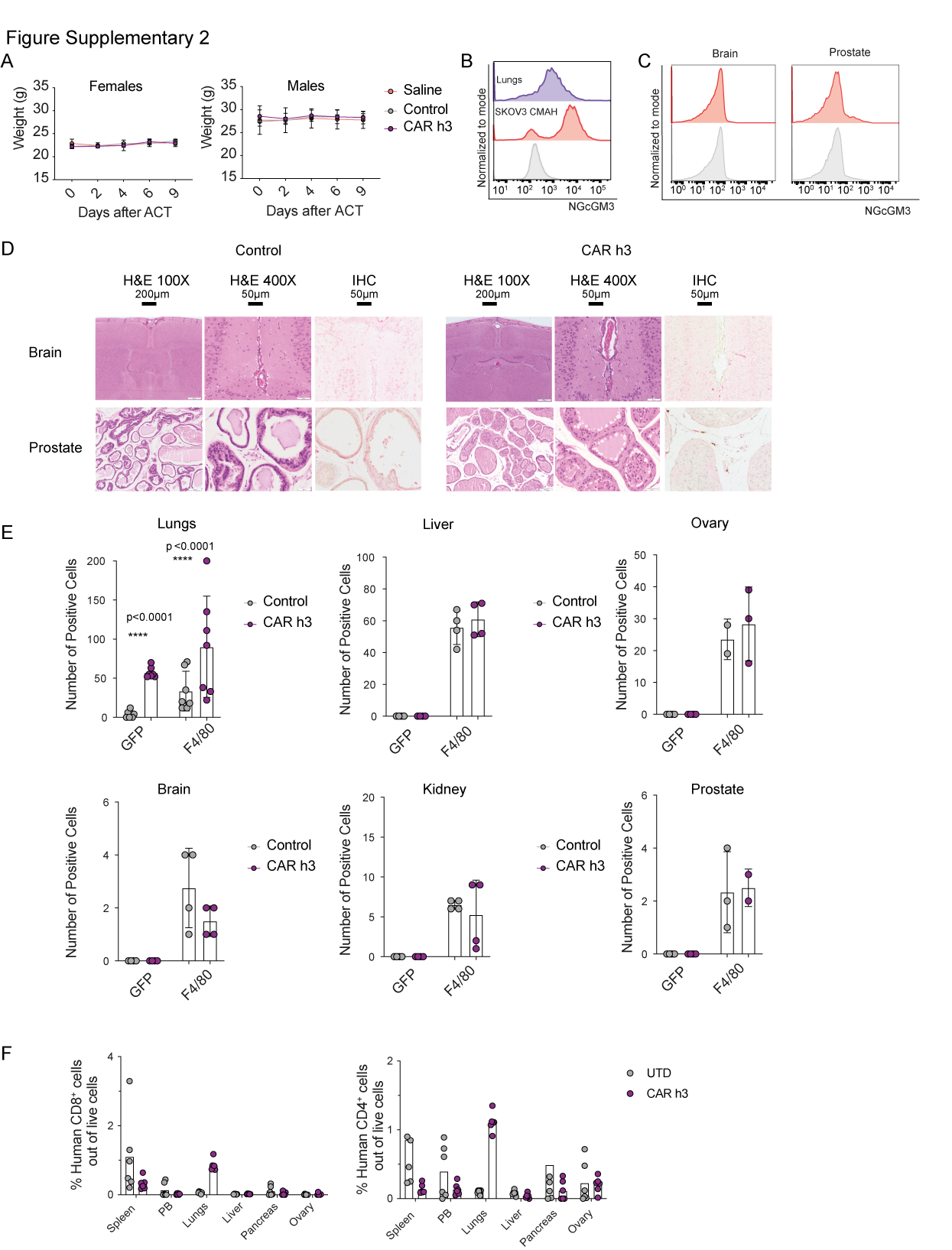

Supplement: Supplementary Figure 2 — (A) Weight of female and male mice over time for in vivo toxicity study (treated as per Figure 3A). (B) Flow cytometric analysis to evaluate NGcGM3 expression by 14F7 mAb staining of dissociated lungs (in purple) and SKOV3 CMAH cells (in red) compared to control (secondary Ab alone, in grey). (C) Flow cytometric analysis to evaluate NGcGM3 expression by 14F7 mAb staining for different dissociated organs (in red) compared to control (secondary Ab alone, in grey). (D) Histopathology of the organs [treated as per Figure 3A): H&E = hematoxylin and eosin staining at 100X and 400X magnification; IHC = immunohistochemistry to detect GFP+ T cells (brown, indicated with arrow)]. (E) Quantification of GFP+ and F4/80+ cells in different organs evaluated by IHC (treated as per Figure 3A). (F) Flowcytometric analysis of dissociated organs and peripheral blood (mice treated as per Figure 3A) to detect human CD8+ (left) and CD4+ (right) T cells. Shown is average ± sem (A), ± SD (E). Statistical analysis unpaired two-tailed t test (E). (****p< 0.0001). [file Image_2.tif]
